# P375: Effective processing of reusable dispensers for surface disinfection tissues – the devil is in the details

**DOI:** 10.1186/2047-2994-2-S1-P375

**Published:** 2013-06-20

**Authors:** G Kampf, C Ostermeyer

**Affiliations:** 1 Bode Science Center, Bode Chemie GmbH, Hamburg, Germany; 2 Institute for Hygiene and Environmental Medicine, Ernst-Moritz-Arndt University, Greifswald, Germany; 3 Microbiology, Bode Chemie GmbH, Hamburg, Germany

## Introduction

Surface disinfectant solution based on surface-active ingredients such as quaternary ammonium compounds, alkylamines or glucoprotamin which are prepared in reusable tissue dispensers may contaminate heavily with gram-negative bacterial species if the dispensers are not processed adequately.

## Objectives

We determined the efficacy of various manual and automatic procedures on contaminated dispensers with the aim to prevent re-contamination of the disinfectant solution over 28 days.

## Methods

Three manual and three automatic procedures were evaluated with at least 3 dispensers per procedure. Most experiments were done with contaminated dispensers from healthcare facilities. In addition, some experiments were done with new dispensers which were freshly contaminated with contaminated solution from a clinical dispenser. After processing, the dispenser was refilled with a surface disinfectant based on benzalkonium chloride (Mikrobac forte, 0.5%), the new tissue role inserted and the filled dispenser left at room temperature. Disinfectant samples were taken after 7, 14, 21 and 28 days. The number of CFU per mL was determined by serial dilution using standard microbiological techniques.

## Results

Use of an alcohol-based surface disinfectant for both cleaning and a separate disinfection step did not prevent recontamination over 4 weeks. A manual procedure using a biofilm-active cleaner followed by an alcohol-based surface disinfectant, however, was effective. Use of an oxygen-releasing disinfectant cleaner for 1 h was also effective. All three automatic procedures were effective as long as the minimum temperature was 60°C and the process time at least 5 min irrespective of using a cleaning agent or not.

## Conclusion

Some commonly recommended procedures for dispenser processing do not prevent recontamination of the disinfectant solution when it is based on surface-active ingredients. Manual procedures with a real cleaning step and some automatic procedures were effective. It becomes evident that processing of reusable dispensers for surface disinfectant tissues requires more effort than commonly thought if the real contamination found in clinical practice should be controlled and if the active ingredients of surface disinfectants are only surface-active substances.

## Disclosure of interest

G. Kampf Employee of Bode Chemie GmbH, Hamburg, Germany, C. Ostermeyer Employee of Bode Chemie GmbH, Hamburg, Germany

